# Study on brain functional networks and cortical morphological structures in patients with primary insomnia

**DOI:** 10.3389/fneur.2026.1770086

**Published:** 2026-02-27

**Authors:** Xiaoyue Jin, Xiaoyang Wang, Kuihua Wang, Yuning Lin, Xiaoling Duan, Shangwen Xu, Xiaoping Cui, Hui Li

**Affiliations:** 1 900th Hospital of PLA Joint Logistic Support Force, Fuzhou, China; 2 Fuzong Clinical Medical College of Fujian Medical University, Fuzhou, China; 3 Department of Medical Imaging, Xiamen Xianyue Hospital, Xianyue Hospital Affiliated with Xiamen Medical College, Fujian Psychiatric Center, Fujian Clinical Research Center for Mental Disorders, Xiamen, China; 4 Fuzong Teaching Hospital of Fujian University of Traditional Chinese Medicine (900th Hospital), Fuzhou, China; 5 Department of Radiology, The Second Affiliated Hospital of Fujian Traditional Chinese Medical University, Fuzhou, China

**Keywords:** central executive network, default mode network, hyperarousal, primary insomnia, surface-based morphometry, voxel-wise degree centrality

## Abstract

**Introduction:**

The prevalence of primary insomnia (PI) has been increasing year by year. This study aims to explore its neurobiological mechanisms and provide neuroimaging evidence for clinical diagnosis and treatment.

**Methods:**

A total of 33 patients with PI and 25 healthy controls were recruited. All participants completed assessments of sleep status, cognitive function, and evaluations using neuropsychological scales. Voxel-based degree centrality (DC) analysis was used to identify abnormal functional network hubs, and differentially activated brain regions were further used as seed points for whole-brain functional connectivity (FC) analysis. Surface-based morphometry (SBM) was employed to analyze cortical morphological parameters. Additionally, DC values, FC values, and cortical morphological parameters of the differentially activated brain regions were extracted from the PI group for the differentially activated brain regions, and further correlation analyses were performed with neuropsychological scale scores.

**Results:**

In patients with PI, the DC of the bilateral precuneus/posterior cingulate gyrus was increased, while the DC of brain regions related to the right parietal lobe was decreased; FC was reduced in some brain regions; the cortex of the left frontoparietal region was thinner with an elevated gyrification index (GI); and the fractal dimension (FD) of the right parieto-occipital cortex/left occipital cortex was decreased.

**Discussion:**

PI patients show abnormal functional hubs (posterior DMN, CEN parietal lobe), reduced FC (CEN-right cerebellum, posterior DMN-occipital lobe), and cortical morphological changes (left frontoparietal thinning, etc.). These neuroimaging indices are correlated with patients’ sleep quality, which can provide a basis for mechanistic research and clinical diagnosis of PI.

## 1 Introduction

Healthy sleep is crucial to physical and mental health, as well as social well-being. In recent years, public awareness of sleep health has been gradually increasing (1, 2). Factors such as the fast pace of modern life, heightened stress, and widespread use of electronic devices have markedly elevated the risk of insomnia. It is estimated that approximately 30–35% of the global population suffers from insomnia (3). Notably, insomnia prevalence varies significantly by gender (higher in females) and age (rising with increasing age) (4). With its prevalence on the rise, insomnia has become an unignorable public health concern.

The primary harms of insomnia include inducing negative emotions (e.g., significantly increasing the risk of depression) (5, 6) and impairing cognitive function (7). Additionally, chronic sleep deprivation is associated with a higher risk of multiple diseases, such as cardiovascular diseases (e.g., coronary heart disease), cerebrovascular diseases, diabetes, and obesity (8–10). This imposes a substantial disease burden on both individuals and society. Given these impacts, early identification and intervention for insomnia are vital to improving patients’ quality of life, reducing the risk of comorbid diseases, and alleviating the social burden.

Currently, the diagnosis of primary insomnia primarily relies on the *Diagnostic and Statistical Manual of Mental Disorders, Fifth Edition* (DSM-5) (11), while the Insomnia Severity Index (ISI) is a commonly used self-report scale to assess symptom severity (12). Studies have shown that an ISI score of ≥8 indicates clinically significant insomnia (13). However, diagnoses based solely on patients’ subjective symptoms have inherent limitations. Against the backdrop of rising insomnia prevalence, further exploration of its neuropathological mechanisms is urgently needed to improve diagnostic accuracy and optimize treatment efficacy.

Research indicates that the pathophysiological mechanisms of PI involve abnormalities in sleep–wake rhythm regulation and hyperarousal states (14). Immune and metabolic factors (15) as well as electroencephalographic features are also involved (16). Functional magnetic resonance imaging (fMRI)—particularly resting-state fMRI (rs-fMRI)—has provided new methodological support for investigating neuropsychiatric disorders such as insomnia (17). Functional connectivity (FC) analysis has revealed abnormal connectivity in brain regions related to sleep–wake regulation in PI patients (18). Voxel-wise degree centrality (DC) can effectively identify key hub nodes in brain networks at the voxel level, serving as a “detector of functional hubs” (19) and exhibiting high test–retest reliability (20). Thus, DC can reflect the importance of hub nodes in brain networks and their changing characteristics at the voxel level. To date, however, no stable abnormal hub regions have been identified in PI research.

In brain structural studies, voxel-based morphometry (VBM) has detected abnormal gray matter volume (GMV) in PI patients, but results remain inconsistent (21). In contrast, surface-based morphometry (SBM) has advantages in measuring cortical characteristics, including cortical thickness and cortical folding degree (22). Preliminary SBM studies suggest that PI may be associated with cortical thickening (23), and that circadian rhythm-related rapid eye movement (REM) sleep regulation exerts a protective effect against age-related brain structural changes (24). Nevertheless, research on this topic remains limited.

While previous studies have reported abnormal brain functional networks and structural microalterations in PI patients, most of these studies are single-modality studies. Thus, the present study aimed to integrate rs-fMRI and 3D T1WI data, applying voxel-wise DC and SBM approaches to examine PI patients who met clinical diagnostic criteria (ISI score ≥8). It focused on alterations in functional hubs and cortical structures at the whole-brain level, and explores the relationships between these abnormal alterations and sleep quality, cognitive function, and emotional states. The ultimate goal is to comprehensively reveal the brain functional and structural alterations associated with PI, elucidate their potential neurobiological mechanisms.

## 2 Materials and methods

### 2.1 Study participants

PI patients who visited the neurology outpatient clinic of our hospital between February 2023 and August 2024 were screened by two neurologists with 15 years of experience using mental and sleep-related clinical interviews. A total of 33 right-handed patients who met the DSM-5 criteria for PI and had an ISI score ≥8 were finally included in the PI group. All patients reported difficulty falling asleep, early awakening, and difficulty maintaining sleep, with an disease duration of > 3 months. Exclusion criteria were as follows: (1) major physical illnesses (cardiovascular, central nervous system, endocrine system, etc.); (2) current or past history of mental disorders; (3) other sleep disorders (e.g., sleep apnoea syndrome, restless legs syndrome); (4) contraindications for MRI scanning; (5) use of medications affecting sleep or the central nervous system within the past month; (6) history of head trauma; (7) history of substance abuse. Additionally, a recruitment poster was posted online to enroll 25 right-handed healthy volunteers matched for age, gender, and years of education. The healthy control (HC) group had no history of mental disorders or sleep disorders and an ISI score ≤7. This study has been approved by the Ethics Committee of the 900th Hospital of the Chinese People’s Liberation Army Joint Logistic Support Force (Ethics Approval Number: 2022–060). All participants provided written informed consent after fully understanding the study protocol prior to participation.

### 2.2 Clinical assessment

Each participant underwent the following assessments: (1) ISI to assess the severity of insomnia symptoms (25), with an ISI score <8 indicating non-clinically significant insomnia; (2) Pittsburgh Sleep Quality Index (PSQI) to assess overall sleep quality over the past month, with a score > 5 indicating poor sleep quality (26); (3) Epworth Sleepiness Scale (ESS) to comprehensively assess daytime sleepiness in various environments (27), with an ESS score ≥ 9 indicating sleepiness; (4) Hamilton Depression Rating Scale (HAMD-24) and Hamilton Anxiety Rating Scale (HAMA) to assess participants’ mood status; (5) Montreal Cognitive Assessment (MoCA) and Mini-Mental State Examination (MMSE) to assess overall cognitive function levels, with MoCA scores <26 and MMSE scores <27 (28) indicating cognitive impairment.

### 2.3 MRI

All subjects underwent cranial magnetic resonance imaging (MRI) scans at the Imaging Center of our hospital. Before the examination, contraindications to magnetic resonance imaging such as intracorporeal implanted metal foreign bodies and claustrophobia were excluded. Participants were instructed to lie flat with their shoulders close to the coil, and earplugs were provided to mitigate scanner noise. A wedge-shaped sponge pad was used to fix the head to minimize head movement. Participants were instructed to keep their eyes closed, remain awake, keep their head still, and avoid engaging in any mental activity during the examination. All participants were scanned using a Siemens Magnetom Trio Tim 3.0 T superconducting MRI scanner and a 12-channel head coil. The examination position was supine with the head-first mode, and the body surface landmark was centred at the glabella.

3D T1WI: Sagittal plane, Repetition Time (TR) = 1900 ms, Echo Time (TE) = 2.5 ms, Number of echoes = 1, Matrix = 256 × 256, Field of View (FOV) = 256 mm × 256 mm, Flip Angle (FA) = 9°, Number of slices = 176, slice thickness = 1.0 mm, slice gap = 0.5 mm, scan time = 4 min 26 s.

Resting-State fMRI: Axial, TR = 2,000 ms, TE = 30 ms, matrix = 64 × 64, FOV = 240 mm × 240 mm, FA = 90°, Number of slices = 33, slice thickness = 4.0 mm, slice gap = 0.8 mm, Voxel Size = 3.8 mm × 3.8 mm × 3.8 mm, scan time = 8 min 8 s, with 240 time points acquired.

### 2.4 Image data preprocessing

#### 2.4.1 rs-fMRI data preprocessing

The DC method was employed to analyze the collected rs-fMRI image data. Raw data preprocessing and DC value calculation were performed using the Resting State Functional Data Processing Assistant Software (DPABI_V8.2) on the Matlab 2022a platform. The specific steps are as follows: (1) Image format conversion; (2) Removal of the first 10 time points to stabilize the magnetic field; (3) Time series correction; (4) Head motion correction: exclusion of participants with head motion translation >2.5 mm or rotation >2.5° to mitigate motion artefacts; (5) Removal of covariates such as cerebrospinal fluid, white matter, whole-brain average signal, and head motion parameters; (6) Filtering using a frequency bandwidth of 0.01–0.08 Hz; (7) Spatial standardization; (8) Spatial smoothing using a 6 mm Gaussian smoothing kernel; (9) Construction of the human brain functional connectome: Calculate the Pearson correlation coefficient matrix of whole-brain voxel-to-voxel time series within the default brain template in DPARSF; (10) DC calculation: A threshold ( $r_{0}$ > 0.25) (20) was set to construct a binary functional connectivity matrix, calculate the total sum of significant correlation weights DC for each node, normalised by dividing by the whole-brain mean DC to generate standardized DC values, and finally use Fisher *Z*-value conversion to obtain the *Z*-normalised DC distribution map of the brain FC network for each subject.

We used the DPARSF software package to perform seed-based FC analysis. Seed regions were selected from brain areas with abnormal activation (brain regions with significant differences in between the PI group and the HC group). Subsequently, by calculating the positive and negative correlations between the seed point and other brain voxels, FC maps were generated. Finally, Fisher transformation was used to convert the correlation coefficient map into a Z-score-normalised FC distribution map.

#### 2.4.2 3D-T1WI data preprocessing

The collected 3D-T1WI image data were analysed using the SBM method. The raw image data were preprocessed on the Matlab 2022a platform using the CAT 12 toolkit of SPM12, with the following steps: (1) Image format conversion; (2) Spatial standardization: alignment of participants’ brain images to the MNI-152 2 mm standard brain template space; (3) Brain tissue segmentation (gray matter, white matter, and cerebrospinal fluid), followed by extraction of cortical parameters (thickness, folding degree [FD], gyrification index [GI], sulcal depth); (4) Smoothing: a 15 mm full-width at half-maximum (FWHM) Gaussian smoothing kernel was applied to cortical thickness maps, and a 25 mm FWHM Gaussian smoothing kernel was applied to other parameter maps.

### 2.5 Statistical analysis

#### 2.5.1 Statistical analysis of general data

SPSS 29.0 statistical software was used to perform statistical analyses of demographic and clinical scale data of the participants. Continuous variables were analysed using independent-samples *t*-test (for normally distributed data) or the Mann–Whitney *U* test (for non-normally distributed data); categorical variables were analysed using the chi-square test. A two-tailed *p-*value < 0.05 was considered statistically significant.

#### 2.5.2 Statistical analysis of DC and FC

In the Matlab 2022a environment, statistical analysis was performed using the DPABISF software. Between-group comparisons of DC statistical maps were performed using a two-sample *t*-test, controlling for covariates such as age, gender, and years of education. The test results were corrected using AlphaSim multiple comparisons. Differences were considered statistically significant when the voxel-level *p*-value < 0.05 and the voxel cluster size > 150; for FC statistical maps, between-group comparisons were performed using a two-sample t-test, with results corrected using Gaussian Random Field (GRF) (voxel-level *p*-value < 0.05, cluster-level *p*-value < 0.05). All significant results were anatomically localized using the Automated Anatomical Labeling Atlas 3 (AAL3) (29) for brain regions showing significant differences. The BrainNet Viewer^1^ toolkit was used to visualize the results of DC and FC differences in regions showing significant differences between the two groups, and brain regions with ≥ 20 voxels were included in further analysis.

#### 2.5.3 SBM statistical analysis

In the Matlab 2022a environment, statistical analysis was performed using the CAT 12 toolbox in SPM12 software. Between-group comparisons were conducted using a two-sample *t*-test, with age and gender as covariates, to assess group differences in cortical morphological features. After cluster-level family-wise error (FWE) correction, results were considered statistically significant at a *p*-value < 0.05. The results were visualised using the results presentation module of the CAT 12 toolbox, and the Desikan-Killiany atlas (DK40, 40 cortical regions) was used to anatomically localise the regions of interest (ROIs).

#### 2.5.4 Correlation analysis

Mean values of brain regions showing significant group differences in DC, FC, and various cortical morphological features between the PI group and the HC group were extracted. Subsequent correlation analyses were performed between these mean values and the participants’ neuropsychological scale scores. For normally distributed continuous variables, Pearson correlation coefficients were used, whereas Spearman correlation coefficients were applied for non-normally distributed continuous variables or ordinal variables. Correlation analysis results were corrected for multiple comparisons (adjusted for age and gender as covariates) using the false discovery rate (FDR) method at a threshold of q-value < 0.05. A heatmap was generated to visualize the significant correlation patterns between brain morphological/functional indices and neuropsychological scale scores, with Matlab 2022a used for heatmap construction and visualization. FDR-corrected results with a *q*-value < 0.05 were considered statistically significant.

**Figure 1:**
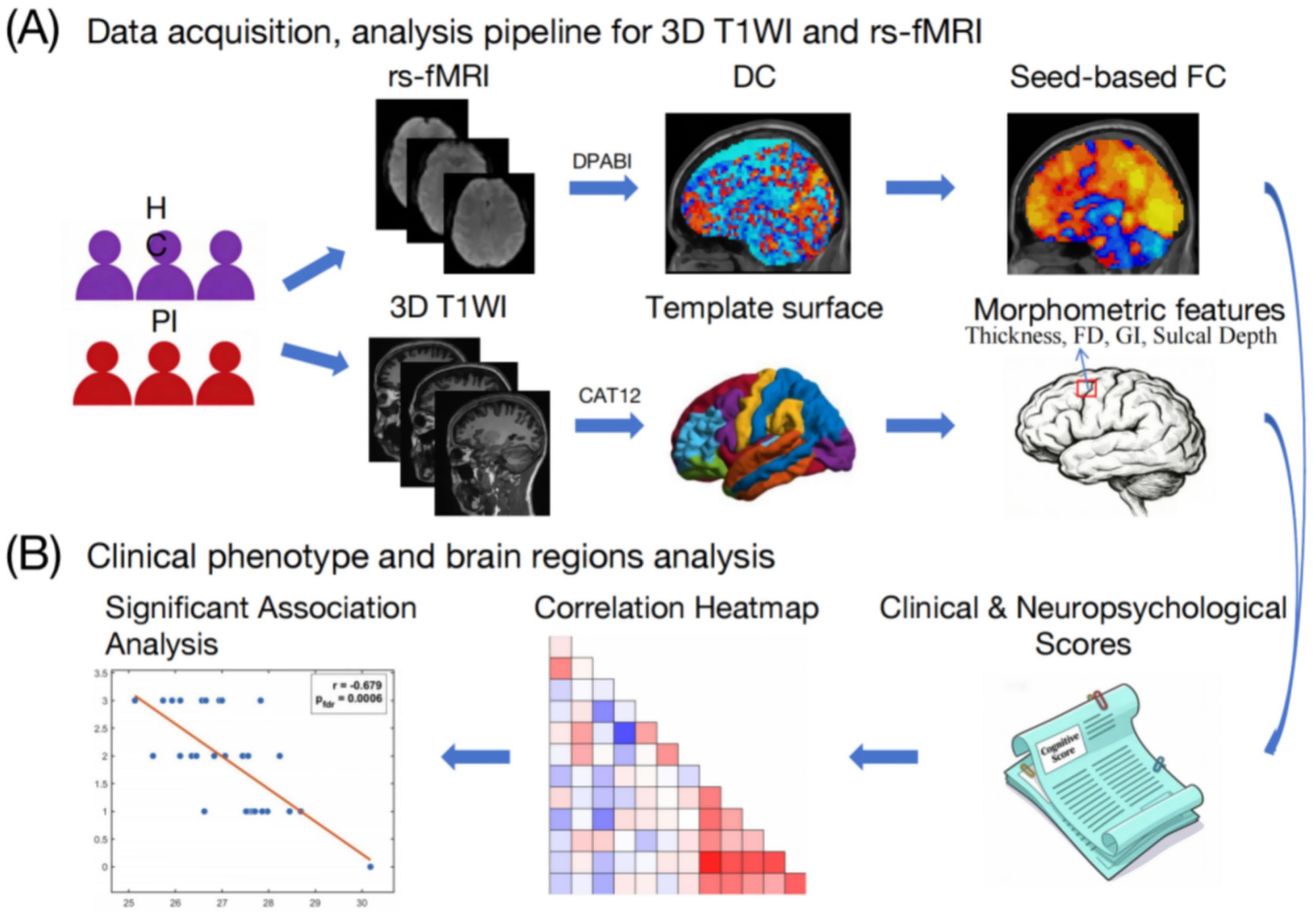
Data acquisition, processing pipeline, and clinical-brain association analysis workflow in the study. **(A)** Schematic of the data acquisition and analysis pipeline for 3D T1-weighted imaging (T1WI) and resting-state functional MRI (rs-fMRI). For rs-fMRI, data were processed using DPABI to compute degree centrality (DC) and seed-based functional connectivity (FC). For 3D T1WI, data were processed using CAT12 to extract morphometric features, including cortical thickness, fractal dimension (FD), gyrification index (GI), and sulcal depth. **(B)** Workflow of clinical phenotype and brain region association analysis, including significant association analysis and correlation heatmap visualization.

The multimodal neuroimaging data acquisition and processing pipeline, as well as the experimental process for association analysis between clinical phenotypes and brain regions in this study, are shown in Figure 1.

## 3 Results

### 3.1 Demographic and clinical characteristics of participants

The demographic and clinical characteristics of the PI group and HC group are presented in Table 1. Statistical analysis revealed no statistically significant differences between the two groups in age, gender, years of education, diabetes, hypertension, smoking status, or alcohol consumption (*p* > 0.05). However, significant group differences were observed in the following indices (*p* < 0.05): total ISI scores; total HAMD and HAMA scores; total PSQI scores and its seven subscale scores; total MoCA scores and the MoCA Visuospatial and Executive Function, Attention, Abstract Thinking, and Delayed Recall subscales; and total MMSE scores and the MMSE Orientation, Attention and Calculation, Delayed Recall, and Language subscales.

**Table 1:** Comparison results of demographic data and clinical scales between the PI group and the HC group.

Item	PI group (*n* = 33)	HC group (*n* = 25)	*p* value
Gender (Male/Female)	14/19	15/10	0.185^a^
Age (years)	51.88 ± 14.51	44.64 ± 15.56	0.074^b^
Years of education (years)	12 (6, 16)	12 (9, 16)	0.595^c^
Diabetes (yes/no)	1/32	3/22	0.417^a^
Hypertension (yes/no)	3/30	6/19	0.235^a^
Smoking (yes/no)	10/23	6/19	0.595^a^
Alcohol consumption (yes/no)	12/21	14/11	0.136^a^
ESS (score)	4 (2, 6)	3 (0, 4)	0.153^c^
ISI (score)	14 (11.5, 18.5)	1 (0, 3)	<0.001^c^
HAMA (score)	16 (7.5, 21.5)	2 (0, 4)	<0.001^c^
HAMD (score)	13 (7.5, 20)	2 (0, 5)	<0.001^c^
PSQI (score)	13.55 ± 3.32	3.28 ± 2.25	<0.001^b^
Sleep quality	2 (2, 3)	1 (0, 1)	<0.001^c^
Sleep onset time	3 (2, 3)	1 (0, 1)	<0.001^c^
Sleep duration	3 (2, 3)	1 (0, 1)	<0.001^c^
Sleep efficiency	3 (1, 3)	0 (0, 0.5)	<0.001^c^
Sleep disturbance	1 (1, 2)	0 (0, 1)	<0.001^c^
Hypnotics	0 (0, 2.5)	0 (0, 0)	0.001^c^
Daytime functioning	2 (1, 3)	0 (0, 1)	<0.001^c^
MMSE (score)	28 (25, 29)	30 (28.5, 30)	0.001^c^
Orientation	10 (9, 10)	10 (10, 10)	0.018^c^
Immediate memory	3 (3, 3)	3 (3, 3)	0.384^c^
Attention and calculation	5 (3.5, 5)	5 (5, 5)	0.018^c^
Delayed recall	2 (1.5, 3)	3 (2, 3)	0.023^c^
Language	9 (8, 9)	9 (9, 9)	0.033^c^
MoCA (score)	23 (18.5, 27.5)	29 (26, 30)	<0.001^c^
Visuospatial and executive function	4 (1.5, 4)	4 (3, 5)	0.034^c^
Naming	3 (2, 3)	3 (2.5, 3)	0.176^c^
Attention	6 (5, 6)	6 (6, 6)	0.003^c^
Language	3 (2, 3)	3 (3, 3)	0.063^c^
Abstract thinking	1 (0, 2)	2 (1, 2)	0.024^c^
Delayed recall	3 (0, 3)	5 (4, 5)	<0.001^c^
Orientation	6 (5, 6)	6 (6, 6)	0.184^c^
Cortical thickness	2.4286 (2.3380, 2.4702)	2.5324 (2.3909, 2.5656)	0.003^c^
Gyrification index	27.1894 ± 1.0128	26.2959 ± 0.6890	<0.001^b^
Fractal dimension	2.6868 (2.6602, 2.7151)	2.7807 (2.7584, 2.8103)	<0.001^c^

^a^Indicates chi-square test; ^b^indicates independent samples *t*-test, expressed as mean ± standard deviation; ^c^indicates Mann–Whitney *U* test, and was expressed as median and interquartile range. *P* value < 0.05 is considered statistically significant.

### 3.2 Resting-state brain network FC analysis

#### 3.2.1 Voxel-based degree centrality (DC) analysis

**Figure 2:**
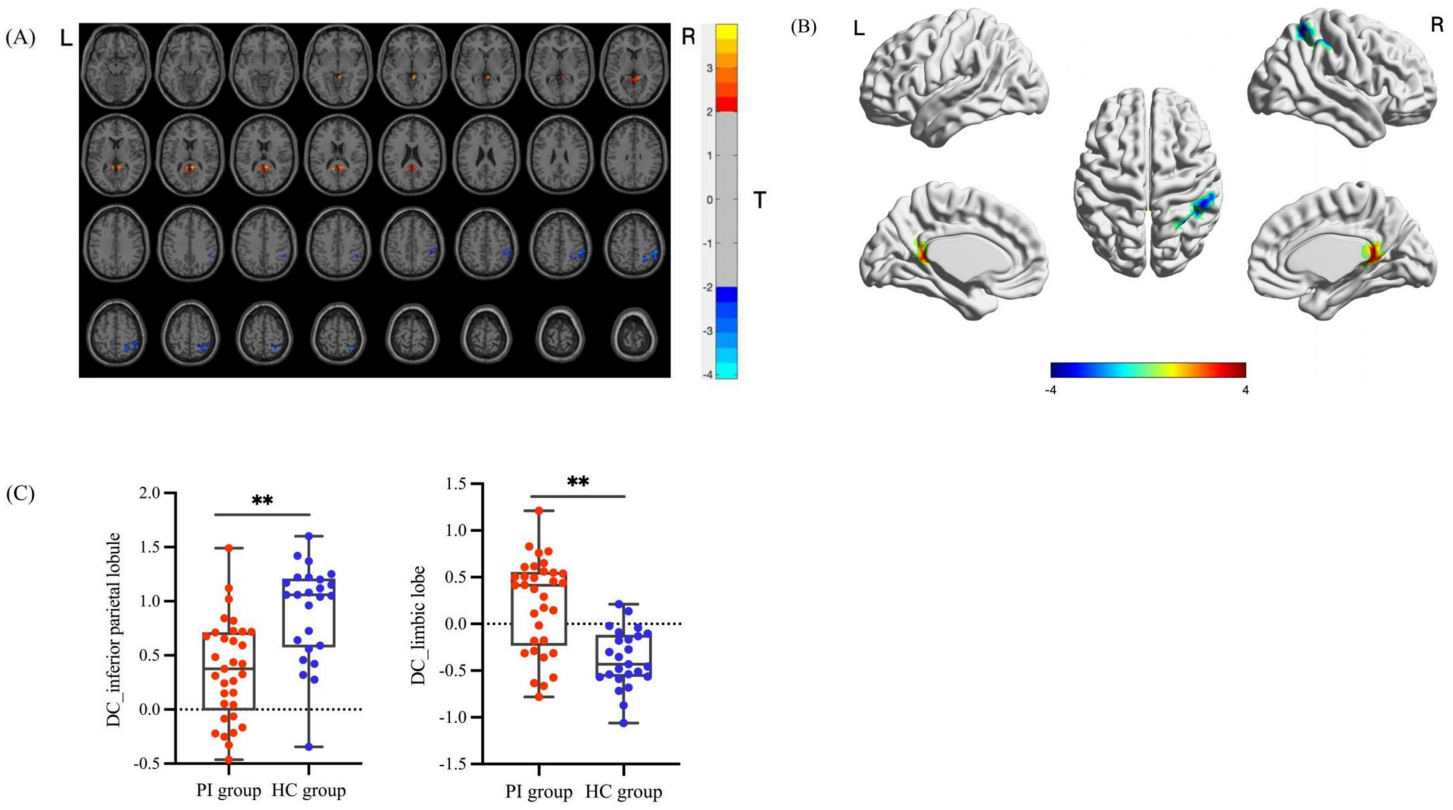
Comparison of brain regions with significant differences in DC between the PI group and the HC group and their DC values. **(A, B)** Brain regions with DC differences between the PI group and the HC group. The colored areas indicate abnormal brain regions. The color-coded values represent *t*-values. **(C)** Comparison of the average DC values of the differential brain regions between groups (**indicates *p* < 0.001).

Compared with the HC group, the regions with significant differences in DC values in the PI group are presented in Table 2 and Figures 2A,B. In the PI group, DC values were significantly increased in the bilateral precuneus/posterior cingulate gyrus; DC values were significantly decreased in the right inferior parietal lobule/superior parietal lobule/supramarginal gyrus (after AlphaSim multiple comparison correction, voxel-level *p* < 0.05, cluster size > 150 voxels). Inter-group comparisons of the mean DC values of two regions showing significant differences (right limbic lobe and right inferior parietal lobule) are illustrated in Figure 2C, with statistically significant differences (*p* < 0.001).

**Table 2:** Brain regions with significant differences in DC between the PI group and the HC group.

Cluster number	Brain regions	Voxel size	MNI peak coordinates (mm)			Peak
			X	Y	Z	
1	Limbic lobe_R	164	6	−45	12	3.9868
	Precuneus_R	44				
	Posterior cingulate gyrus_R	28				
	Precuneus_L	23				
	Posterior cingulate gyrus_L	20				
2	Inferior parietal lobule_R	179	51	−51	57	−4.0925
	Inferior parietal gyrus_R	78				
	Superior parietal lobule_R	63				
	Supramarginal gyrus_R	26				

#### 3.2.2 Seed-based FC analysis

**Figure 3:**
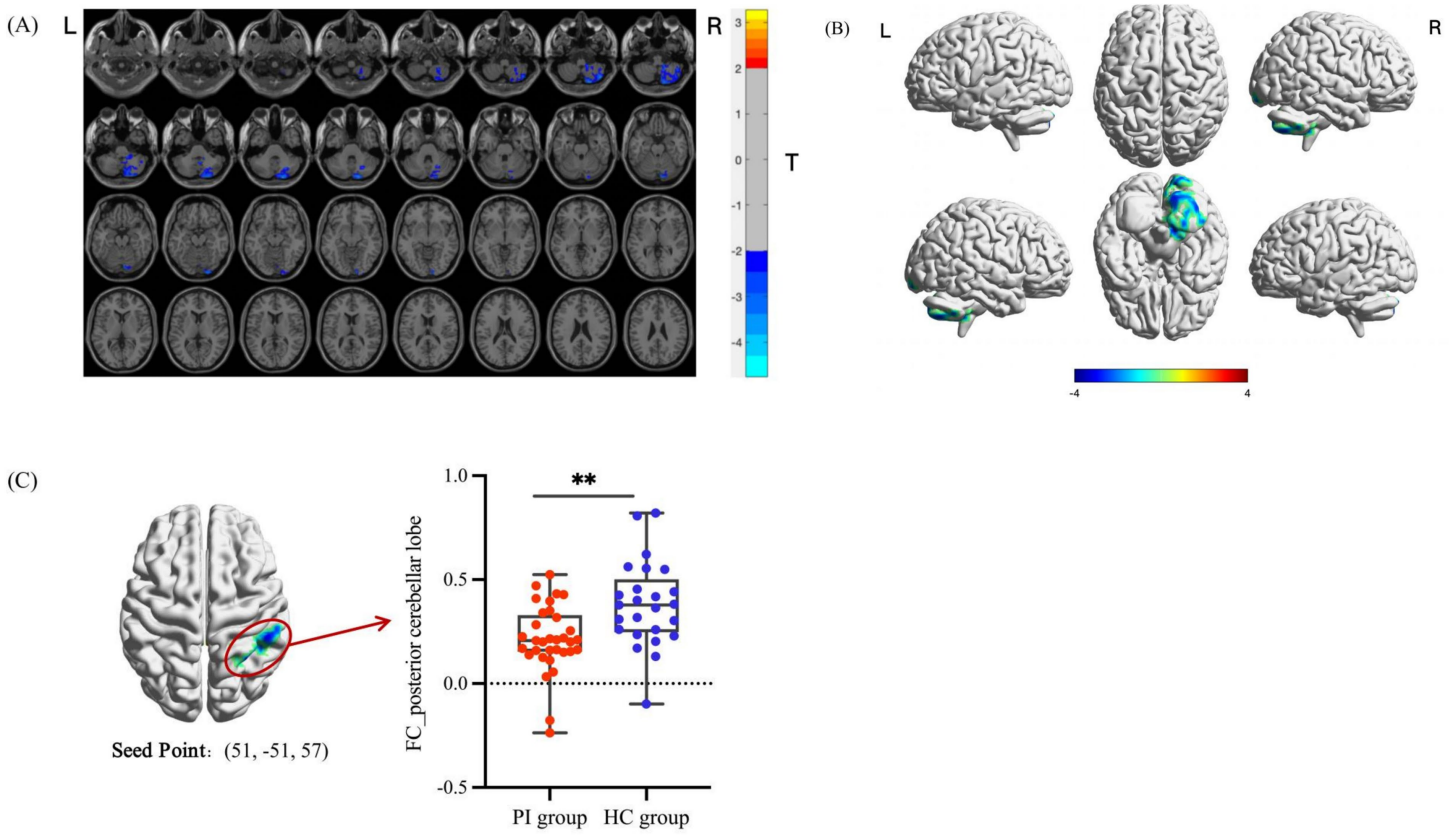
Analysis of differential brain areas in FC with the right inferior parietal lobule as the seed point and intergroup comparison of their FC values. **(A, B)** Brain regions with differences in FC between the PI group and the HC group. The colored areas indicate abnormal brain regions. The color-coded values represent *t*-values. **(C)** Comparison of the average FC values of the differential brain regions between groups (**indicates *p* < 0.001).

**Figure 4:**
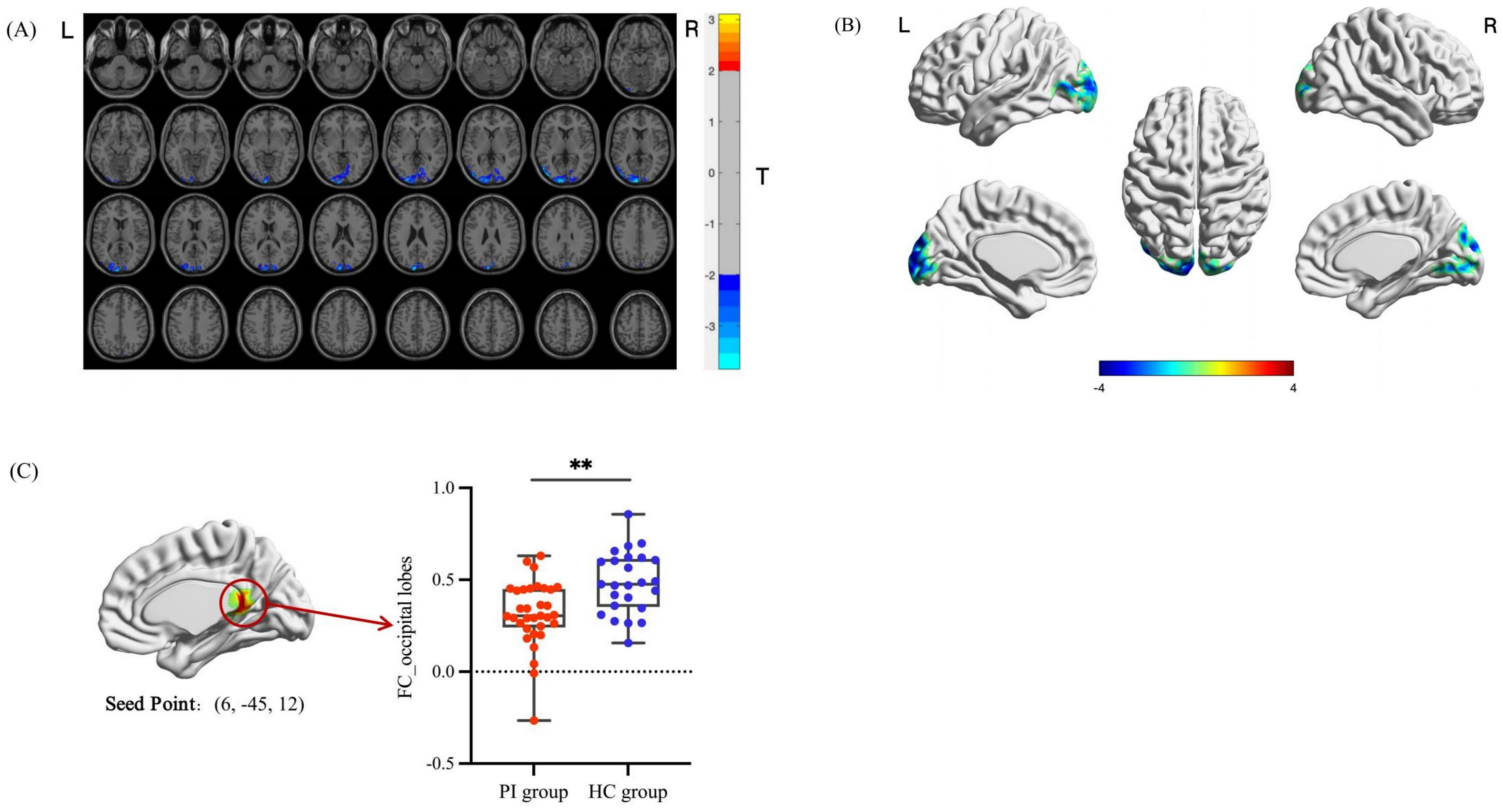
Analysis of differential brain areas in FC with the right limbic lobe as the seed point and intergroup comparison of their FC values. **(A, B)** Brain regions with differences in FC between the PI group and the HC group. The colored areas indicate abnormal brain regions. The color-coded values represent *t*-values. **(C)** Comparison of the average FC values of the differential brain regions between groups (**indicates *p* < 0.001).

The peak brain regions with significant differences identified in the DC analysis were defined as regions of interest (ROIs). Seed-based FC analyses were further performed between these two ROIs and the whole brain, and the results are presented in Table 3 and Figures 3, 4. Compared with the HC group, PI patients exhibited decreased FC between the right inferior parietal lobule and right posterior cerebellar lobe, as well as decreased FC between the right limbic lobe and bilateral occipital lobes (after GRF correction, voxel-level *p* < 0.05, cluster-level *p* < 0.05). No brain regions showing increased FC between the two ROIs and the whole brain were detected. Inter-group comparisons of the mean FC values of the brain regions with significant FC differences are illustrated in Figures 3C, 4C, with statistically significant differences (*p* < 0.001).

**Table 3:** Seed-based FC analysis of brain regions with significant differences between the two groups.

Seed point	Brain regions	Voxel size	MNI peak coordinates (mm)			Peak
			*X*	*Y*	*Z*	
Inferior parietal lobule_R	Posterior cerebellar lobe_R	661	18	−87	33	−3.6478
	Cerebellar Crus 2_R	264				
	Cerebellum 8_R	127				
	Cerebellar Crus 1_R	78				
	Cerebellar 7b_R	57				
	Cerebellar 9_R	49				
Limbic lobe_R	Occipital lobe_bilateral	691	−9	−102	9	−3.8179
	Middle occipital gyrus_L	200				
	Pericalcarine cortex_L	113				
	Superior occipital gyrus_L	74				
	Cuneus_L	61				
	Pericalcarine cortex_R	53				
	Cuneus_R	44				
	Lingual gyrus_R	38				
	Superior occipital gyrus_R	27				

### 3.3 Inter-group comparisons based on cortical surface morphometric measurements

Two-sample *t*-test analyses revealed significant group differences in cortical thickness, FD, and GI across brain regions. However, no significant inter-group differences were observed in sulcal depth.

#### 3.3.1 Changes in cortical thickness

**Figure 5:**
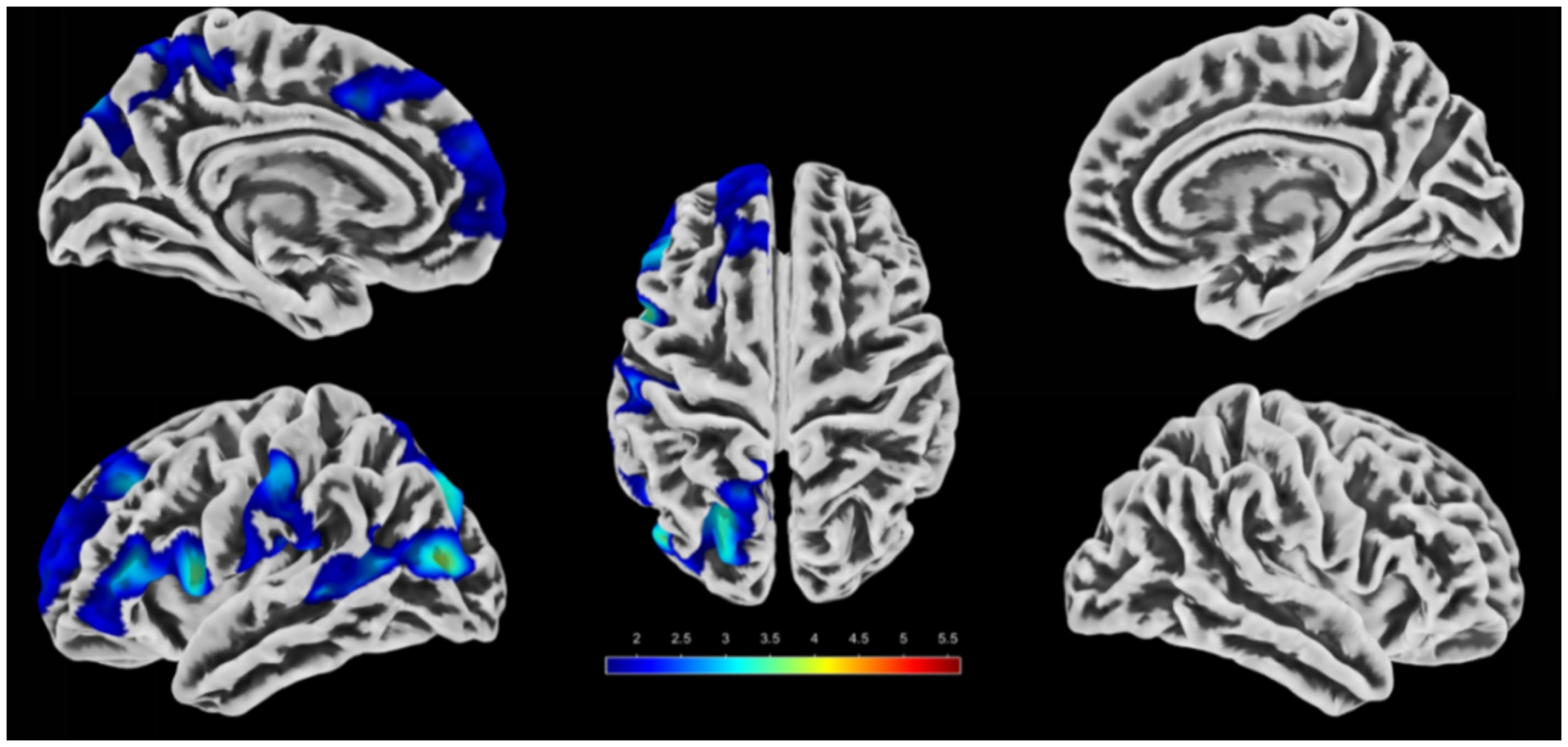
Brain regions with reduced cortical thickness in the PI group compared to the HC group. The colored areas indicate abnormal brain regions. The color-coded values represent *t*-values.

Compared with the HC group, regions with reduced cortical thickness in PI patients are presented in Figure 5 and Table 4. The main involved brain regions included the left superior parietal lobule, left inferior parietal lobule, posterior bank of the left superior temporal sulcus, left precuneus, left paracentral lobule, lateral occipital lobe, left superior temporal gyrus, left supramarginal gyrus, left superior frontal gyrus, anterior part of the left middle frontal gyrus, opercular part of the left inferior frontal gyrus, triangular part of the left inferior frontal gyrus, left precentral gyrus, orbital part of the left inferior frontal gyrus, and left postcentral gyrus (after FWE correction, voxel-level *p* < 0.05, cluster-level *p* < 0.05, cluster size ≥ 945 voxels). No brain regions with significantly increased cortical thickness were observed in the PI group.

**Table 4:** Brain regions with significant differences in cerebral cortical thickness when comparing the PI group and the HC group.

Cluster no.	Differential brain region (Desikan–Killiany, DK40)	Hemisphere	Cluster size	MNI peak coordinates			*P* value
				*X*	*Y*	*Z*	
1	Superiorparietal 29%	L	2,527	−38	−79	11	<0.001
	Inferiorparietal 24%						
	Bankssts 17%						
	Precuneus 12%						
	Paracentral 5%						
	Lateraloccipital 5%						
	Superiortemporal 4%						
	Supramarginal 3%						
2	Superiorfrontal 72%	L	1,211	−19	33	39	0.009
	Rostralmiddlefrontal 22%						
3	Parsopercularis 35%	L	991	−51	10	7	0.036
	Parstriangularis 26%						
	Rostralmiddlefrontal 19%						
	Precentral 12%						
	Parsorbitalis 7%						
4	Postcentral 58%	L	945	−56	−22	44	0.048
	Supramarginal 41%						

All the above differential brain regions are those with thinner cortical thickness in the PI group compared with the HC group; there are no brain regions with significantly thicker cortical thickness in the PI group than in the HC group. MNI, Montreal Neurological Institute.

#### 3.3.2 Changes in cortical fractal dimension

**Figure 6:**
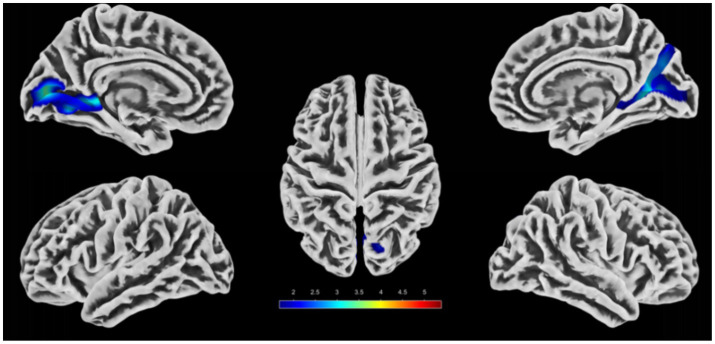
Brain regions with reduced cortical FD in the PI group compared to the HC group. The colored areas indicate abnormal brain regions. The color-coded values represent *t*-values.

Compared with the HC group, regions with decreased cortical FD in PI patients are presented in Figure 6 and Table 5. The main involved brain regions included the bilateral lingual gyri, bilateral pericalcarine cortices, bilateral isthmuses of the cingulate gyrus, bilateral cunei, left parahippocampal gyrus, right precuneus, and right superior parietal area (after FWE correction, voxel-level *p* < 0.05, cluster-level *p* < 0.05, cluster size ≥ 910 voxels). No brain regions with significantly increased cortical FD were observed in the PI group.

**Table 5:** Brain regions with significant differences in fractal dimension of cerebral cortex between PI group and HC group.

Cluster no.	Differential brain region (Desikan–Killiany, DK40)	Hemisphere	Cluster size	MNI peak coordinates			*P* value
				*X*	*Y*	*Z*	
1	Lingual 51%	L	910	−13	−83	7	0.049
	Pericalcarine 30%						
	Isthmuscingulate 12%						
	Cuneus 5%						
	Parahippocampal 2%						
2	Precuneus 28%	R	1,186	27	−63	3	0.008
	Pericalcarine 26%						
	Lingual 18%						
	Cuneus 12%						
	Isthmuscingulate 7%						
	Superiorparietal 6%						

All the above differential brain regions refer to those with decreased cortical fractal dimension in the PI group compared with the HC group; no brain regions with significantly increased cortical fractal dimension in the PI group relative to the HC group were observed. MNI: Montreal Neurological Institute.

#### 3.3.3 Changes in the gyrification index

**Figure 7:**
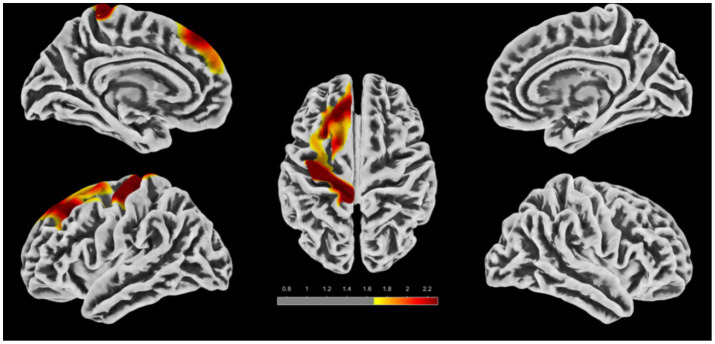
Brain regions with increased GI in the PI group compared to the HC group. The colored areas indicate abnormal brain regions. The color-coded values represent *t*-values.

Compared with the HC group, regions with increased cortical GI in PI patients are presented in Figure 7 and Table 6. The main involved brain regions included the left superior frontal gyrus, left precentral gyrus, caudal part of the left middle frontal gyrus, left postcentral gyrus, anterior part of the left middle frontal gyrus, and left paracentral lobule (after FWE correction, voxel-level *p* < 0.05, cluster-level *p* < 0.05, cluster size ≥ 2,095 voxels). No brain regions with significantly decreased cortical GI were observed in the PI group.

**Table 6:** Brain regions with significant differences in gyrification index between PI group and HC group.

Cluster No.	Differential brain region (Desikan–Killiany, DK40)	Hemisphere	Cluster size	MNI peak coordinates			*P* value
				*X*	*Y*	*Z*	
1	Superiorfrontal 40%	L	2094	−6	−38	68	0.001
	Precentral 31%						
	Caudalmiddlefrontal 10%						
	Postcentral 9%						
	Rostralmiddlefrontal 5%						
	Paracentral 3%						

The above brain regions are those with increased gyrus index in the PI group compared with the HC group; no brain regions with significantly decreased gyrus index in the PI group relative to the HC group were found. MNI: Montreal Neurological Institute.

### 3.4 Correlation analysis

**Figure 8:**
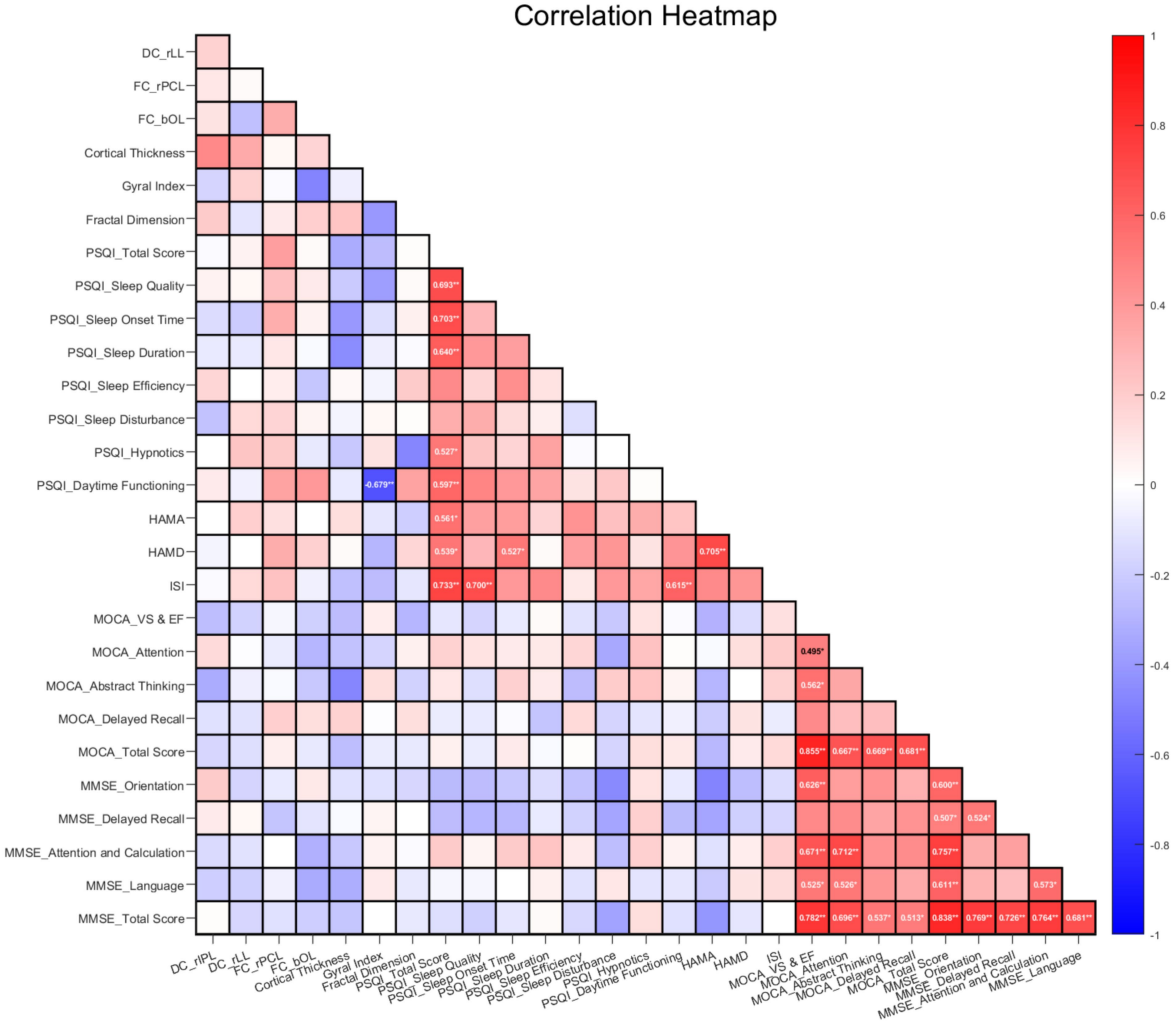
Correlation matrix heatmap of brain functional network indices, cortical morphometric features, and clinical scale scores in the PI group (corrected by FDR). rIPL: right inferior parietal lobule; rLL: right limbic lobe; rPCL: right posterior cerebellar lobe; bOL: bilateral occipital lobes; MoCA_VS & EF: MoCA visuospatial and executive function subscale. The color gradient ranges from blue (negative correlation) to red (positive correlation), with the color bar indicating correlation coefficient (*r*) values. Only associations significant after false discovery rate (FDR) multiple comparison correction (*q* < 0.05) are displayed. Blank or pale-colored cells indicate non-significant correlations (*q* ≥ 0.05). Statistical significance is further marked with * (*p* < 0.05) and ** (*p* < 0.01) for clarity.

After applying false discovery rate (FDR) correction for multiple comparisons (significance threshold: *q* < 0.05), significant correlations were identified in the PI group: cortical gyrification index (GI) was negatively correlated with the PSQI Daytime Functioning subscale score (*r* = −0.679, nominal *p* < 0.001, FDR-corrected *p* < 0.001). No significant correlations were detected in the HC group (all FDR-corrected *p* > 0.05). The detailed correlation coefficients, nominal *p*-values, and FDR-corrected *p*-values for all analyses are summarized in Figure 8 and Table 7.

**Table 7:** Summary of correlations between neuroimaging metrics and clinical scale scores in PI and HC groups: nominal vs. FDR-corrected *P*-values.

Group	Neuroimaging metric	Clinical scale	Nominal *P*-value	FDR-corrected *P*-value	*r*
PI	FC_rPCL	PSQI_Daytime Functioning	0.04	0.20	0.367
	FC_bOL	PSQI_Daytime Functioning	0.03	0.13	0.402
	Cortical Thickness	PSQI_Sleep Onset Time	0.02	0.13	−0.402
	Cortical Thickness	PSQI_Sleep Duration	0.01	0.08	−0.446
	Cortical Thickness	MOCA_Abstract Thinking	0.01	0.06	−0.474
	gyrification index	PSQI_Sleep Quality	0.04	0.18	−0.379
	gyrification index	PSQI_Daytime Functioning	<0.001	<0.001	−0.679
	Fractal Dimension	PSQI_Daytime Functioning	0.04	0.20	0.365
HC	DC_rIPL	PSQI_Sleep Duration	0.04	0.28	−0.422
	DC_rIPL	MOCA_Delayed Recall	0.03	0.21	−0.456
	DC_rLL	PSQI_Sleep Duration	0.03	0.24	−0.441
	DC_rLL	PSQI_Daytime Functioning	0.04	0.25	−0.435
	FC_rPCL	HAMA	0.03	0.21	−0.459
	FC_rPCL	MOCA_Attention	0.02	0.17	−0.481
	Cortical Thickness	PSQI_Sleep Disturbance	0.01	0.11	−0.515

FDR (false discovery rate) correction was applied for multiple comparisons, with *q* < 0.05 as the significance threshold. *P* < 0.05 (FDR-corrected) indicates statistical significance. rIPL, right inferior parietal lobule; rLL, right limbic lobe; rPCL, right posterior cerebellar lobe; bOL, bilateral occipital lobes.

## 4 Discussion

Our findings demonstrate that the bilateral precuneus/posterior cingulate gyrus is one of the key hubs in the abnormal functional network of PI patients, exhibiting aberrant activation patterns. As a core node of the posterior Default Mode Network (DMN) (30), this brain region plays a pivotal role in cognitive processes including episodic memory retrieval and self-reflection, with a particular specialization in contextual information processing (31–34). Among the pathological mechanisms underlying PI, the hyperarousal hypothesis is the most widely accepted. This framework posits that difficulties in sleep onset and maintenance are primarily driven by elevated physical or cognitive hyperarousal (35, 36). Cortical hyperarousal is thought to arise from the brain‘s excessive, repetitive focus on negative cognitions—encompassing hyperactive problem-solving processes, recurrent self-reflection, and persistent worry (37, 38). Accordingly, the aberrant activation of the bilateral precuneus/posterior cingulate gyrus in PI patients aligns closely with the hyperarousal hypothesis; this phenomenon may reflect amplified rumination on negative events in PI patients, ultimately precipitating a state of sustained hyperarousal. Previous studies have reported that the glucose metabolic rate in the precuneus/posterior cingulate gyrus of PI patients shows minimal discrepancy between sleep and wakefulness (39). This observation suggests that these brain regions maintain sustained functional activity at the molecular level in insomniacs, even across sleep–wake transitions, thereby providing theoretical evidence for aberrant activation of the posterior DMN in this patient population. Moreover, existing research has demonstrated that excessive DMN activation is associated with emotional dysregulation and enhanced retrieval of negative memories (40). The elevated anxiety and depression scores observed in the PI group in the present study further support this association. Taken together, our results lend further support to the hyperarousal hypothesis of insomnia.

Meanwhile, we identified the right parietal lobe (encompassing the inferior parietal lobule, superior parietal lobule, and supramarginal gyrus) as another critical hub in the abnormal functional network of PI patients. This hub region is primarily part of the Central Executive Network (CEN) and is implicated in a suite of high-order cognitive processes, including working memory, decision-making, and the regulation of goal-directed behaviors (41). Within the parietal lobe, subregions affiliated with the DMN—such as the precuneus investigated in the present study—directly subserve memory retrieval functions. In contrast, parietal regions outside the DMN are more involved in post-retrieval memory processes, particularly memory-based decision-making (31). In this study, we observed a significant reduction in DC values of the right parietal lobe among whole-brain functional network nodes in PI patients. Previous research has demonstrated that insomniac patients exhibit diminished functional activity in brain regions linked to executive working memory tasks (e.g., the bilateral inferior parietal lobule) (42), accompanied by significantly decreased amplitude of low-frequency fluctuations (ALFF) in the inferior parietal lobule (43) and right supramarginal gyrus (44). These findings corroborate our results, suggesting that CEN-associated parietal regions may undergo functional hypoactivity during episodic memory-based decision-making in PI patients. This functional suppression may be linked to the regulation of hyperarousal in insomniac populations; however, direct empirical evidence supporting a putative “protective effect” of this suppression remains elusive.

In the seed-based FC analysis, we observed reduced FC between the bilateral posterior cingulate gyrus/precuneus and specific subregions of the bilateral occipital lobe in PI patients. The occipital lobe is classically implicated in visual processing, spanning from basic sensory encoding of visual stimuli to higher-order abstract visual functions (e.g., dream imagery). Previous studies have also documented that aberrant neural activity within the occipital lobe may correlate with disruptions in sleep architecture and circadian rhythm regulation (45). Notably, all participants in the present study were instructed to keep their eyes closed throughout the MRI scanning session, thereby eliminating confounding effects of exogenous simple visual stimuli. Recent research has reported that patients with objectively short sleep duration exhibit significantly reduced FC between the left inferior occipital gyrus and the bilateral occipital, parietal, and temporal lobes (46). Furthermore, sleep deprivation studies have demonstrated diminished brain activation in visual-associated cortical regions, including the occipital cortex, lingual gyrus, and fusiform gyrus (47). Building on our findings, the posterior DMN is likely involved in the hyperarousal pathway of insomniac patients—consistent with the elevated DC values of the bilateral precuneus/posterior cingulate gyrus reported in Section 3.2. The reduced FC between the posterior DMN and occipital cortex probably reflects impaired functional interaction between these two brain regions. One plausible interpretation is that this FC reduction may serve to modulate excessive activation of complex abstract visual functions in PI patients, which aligns well with the hyperarousal hypothesis. However, this interpretation remains merely speculative at present: in the absence of objective sleep monitoring data (e.g., polysomnography-recorded sleep stages and parameters) or intervention-based validation (e.g., FC alterations following effective insomnia treatment), we cannot definitively establish this as a bona fide “protective mechanism.”

Additionally, we observed a significant reduction in seed-based FC between the right parietal lobe and multiple subregions of the right posterior cerebellum in PI patients. Although the cerebellum is classically known for its core role in modulating motor coordination and balance, it is also implicated in the regulation of sleep–wake states and sleep rhythm homeostasis (48). The cerebellum establishes tight bidirectional anatomical and functional connections with the cerebral cortex via the thalamus, pontomesencephalic junction, and inferior olivary nucleus. Oscillatory activity within this circuit is highly sensitive to fluctuations in sleep–wake states (49–51). Animal studies have further demonstrated that lesions targeting the cerebellar vermis and cerebellar hemispheres in cats prolong the average duration of non-rapid eye movement (NREM) sleep and total rapid eye movement (REM) sleep duration, while concurrently reducing the frequency of sleep–wake cycle transitions (52, 53). Collectively, these lines of evidence suggest that the reduced FC between the right posterior cerebellum and right parietal cortex may be linked to dysregulated sleep rhythms in PI patients.

This study demonstrated structural aberrations in the frontoparietal cortex of PI patients, characterized by cortical atrophy in the left frontoparietal region accompanied by increased cortical GI, and reduced cortical FD in partial subregions of the right parietal cortex. Notably, the frontoparietal cortex is a key component of the CEN (54). Cerebral cortical activity serves a pivotal role in the execution of high-order cognitive tasks (55), with the prefrontal cortex (56) and parietal cortex (57, 58) being particularly critical to these processes. Previous studies have indicated that reduced GMV in the left precuneus and bilateral inferior parietal cortical atrophy may act as potential biomarkers for the progression from amnestic mild cognitive impairment (aMCI) to Alzheimer’s disease (AD) (59). Furthermore, a bidirectional interplay has been proposed to exist between insomnia and cognitive decline (60)—chronic insomnia could accelerate cognitive impairment, while incipient cognitive deficits might also disrupt sleep regulatory mechanisms.

However, in the present study, although the PI group exhibited global cognitive impairment, the exact relationship between this deficit and frontoparietal cortical structural damage remains unestablished. Notably, we further identified that PI patients had elevated GI in the left frontoparietal cortex, and this index was significantly negatively correlated with the PSQI Daytime Functioning subscale score after FDR multiple comparison correction (*q* < 0.05)—specifically, higher GI values correlated with lower PSQI Daytime Functioning subscale scores, implying alleviated daytime functional impairment. This finding raises the intriguing possibility that increased cortical folding complexity in this region may reflect a form of beneficial neuroplasticity or neural reserve mechanism in PI patients, which could help buffer daytime functional decline induced by sleep disturbances. This perspective deviates from the traditional view that brain structural abnormalities are simply equated with impaired neural function, suggesting that compensatory or adaptive structural reorganization may occur in the brain during the pathological progression of insomnia. Therefore, future studies focusing on subgroup stratification of insomnia patients may help better elucidate the underlying pathological mechanisms linking frontoparietal structural abnormalities to cognitive dysfunction. Moreover, longitudinal studies combining structural imaging and functional assessments are warranted to verify the existence of this adaptive neuroplasticity and clarify its role in the long-term trajectory of daytime functional status in PI patients.

Meanwhile, this study found reduced cortical FD in the occipital lobe (pericalcarine cortex/cuneus/lingual gyrus) and partial limbic lobe subregions (isthmus of the cingulate gyrus/parahippocampal gyrus) of PI patients. Notably, these regions partially overlap with those exhibiting reduced FC between the posterior DMN and occipital lobe, as reported earlier. This convergence may underpin a structural substrate for the observed functional abnormalities, further reinforcing the critical regulatory role of aberrant functional activity in these regions in the hyperarousal pathophysiology of insomnia. Separately, we observed cortical atrophy in the left temporal lobe of PI patients, specifically involving the posterior bank of the superior temporal sulcus and superior temporal gyrus. Previous studies have documented decreased ALFF in the superior temporal gyrus (44) and reduced GMV in the central and lateral temporal lobes of PI patients (61). Conversely, some researchers have proposed that excessive temporal lobe activation may constitute a core factor undermining insomniac patients’ ability to initiate or maintain sleep (62). Although no abnormal functional activity was detected in the temporal lobe of PI patients in our study, cortical thinning in the posterior bank of the left superior temporal sulcus and left superior temporal gyrus may be linked to sleep onset or maintenance difficulties. Further studies are warranted to elucidate this potential association.

This study has several limitations that should be acknowledged. First, the sample size included in this study is relatively modest, which compromises both statistical power and the generalizability of the findings to a broader, more heterogeneous population of insomniac patients—including those with distinct insomnia subtypes (e.g., onset insomnia, maintenance insomnia) and varying disease durations. Second, our study relied solely on subjective sleep assessment tools, which are susceptible to recall bias and subjective reporting discrepancies. Future studies should therefore integrate objective sleep metrics, such as polysomnography (PSG)-derived parameters (e.g., sleep efficiency, arousal index, sleep stage distribution), to conduct a holistic assessment of patients’ sleep physiology. Third, this study is limited by the lack of white matter structural analysis, as we only investigated gray matter cortical parameters. In line with Hidese et al. (63), who reported that poorer sleep quality correlates with reduced white matter integrity in healthy adults. Thus, our findings may not capture the full spectrum of brain structural abnormalities in PI, and future research should integrate gray and white matter assessments to address this gap. Fourth, the cross-sectional study design precludes the establishment of causal relationships between PI, brain structural/functional abnormalities, and cognitive-emotional disturbances. Moving forward, longitudinal cohort studies stratified by insomnia subtypes, disease severity, or cognitive status are warranted to elucidate the underlying biological mechanisms and directional causality of these associations.

## 5 Conclusion

PI patients exhibit distinct functional and structural brain abnormalities. Functionally, there is elevated DC in the posterior DMN (bilateral precuneus/posterior cingulate gyrus) and reduced DC in the parietal lobe component of the CEN (right inferior parietal lobule). Structurally, PI patients present with cortical thinning in the left frontoparietal cortex and an increased cortical GI. Among these abnormalities, reduced FC between the right parietal lobe of the CEN and the right posterior cerebellum is associated with sleep–wake disturbances. Meanwhile, diminished FC between the posterior DMN and the occipital lobe might be involved in the regulation of hyperarousal, though its putative protective role remains unconfirmed. Notably, the increased GI in the left frontoparietal cortex of PI patients—which reflects enhanced cortical folding complexity—may act as a form of beneficial neuroplasticity or neural reserve mechanism that buffers the decline in daytime function induced by sleep disorders. Collectively, this study provides valuable multimodal neuroimaging evidence for the neurobiological mechanisms underlying primary insomnia.

## Data Availability

The raw data supporting the conclusions of this article will be made available by the authors, without undue reservation.

## Footnotes

1
http://www.nitrc.org/projects/bnv/
